# Prevalence and factors associated with self-medication among schoolchildren in the Metropolitan Region of Vitória, Brazil

**DOI:** 10.1590/1980-220X-REEUSP-2025-0395en

**Published:** 2026-03-16

**Authors:** Kamila Rocha Mairink, Fernanda Garcia Gabira Miguez, Carlos Augusto Lopes, Franciele Foschiera Camboin, Nathalia Miguel Teixeira Santana, Nathalia Borba Raposo Pereira, Franciéle Marabotti Costa Leite

**Affiliations:** 1 Universidade Federal do Espírito Santo, Departamento de Enfermagem, Vitória, ES, Brazil.; 2 Universidade Federal do Espírito Santo, Programa de Pós-Graduação em Saúde Coletiva, Vitória, ES, Brazil.; 3 Universidade Federal do Espírito Santo, Programa de Ciências Sociais, Vitória, ES, Brazil.; 4 Universidade Estadual do Oeste do Paraná, Cascavel, PR, Brazil.; 5 Universidade Federal do Espírito Santo, Programa de Pós-Graduação em Psicologia, Vitória, ES, Brazil.

**Keywords:** Adolescent, Adolescent Health, Self Medication, Nonprescription Drugs

## Abstract

**Objective:**

To analyze the prevalence and factors associated with self-medication at some point in life and its current use among high school students in the Greater Vitória Metropolitan Region, Espírito Santo.

**Method:**

This cross-sectional, school-based study was conducted between March and December 2023 with 4,416 students. The outcome was experimentation with and current use of nonprescribed medication. The categorical independent variables were sex, age group, gender identity, race/skin color, socioeconomic status, type of school, current employment or business, religion, and parental marital status. Data were analyzed using Pearson’s chi-square test and Poisson regression.

**Results:**

The prevalence of lifetime self-medication among schoolchildren was 22.7% (95%CI: 21.5–23.9), being more prevalent among females (28.5%; 95%CI: 26.8–30.3). There was a higher prevalence of lifetime self-medication and current self-medication among female students, LGBT+ individuals, those from higher socioeconomic classes, and those with employment.

**Conclusion:**

The findings reinforce the complexity of self-medication among adolescents, which involves not only issues of access to and availability of medications, but also psychosocial and cultural aspects.

## INTRODUCTION

Access to medications has provided society with benefits that include the prevention, maintenance, and recovery of health, contributing, in general, to prolonging human vitality^(1)^. However, despite these undeniable advances, the irrational use of medications constitutes a public health problem, since their inappropriate use results in health risks^(2)^.

According to the World Health Organization, more than half of all medications are dispensed, sold, and prescribed incorrectly. Furthermore, over 50% of all countries fail to implement basic policies to promote their rational use^(3)^. In Brazil, however, the misuse of medication is related to prescriptions not guided by technical and clinical guidelines established by regulatory bodies and scientific societies, inappropriate self-medication, and the wide availability of medications for purchase on the market^(4)^.

According to a survey conducted by the Federal Council of Pharmacy in 2019, 77% of Brazilians self-medicated between July and December. This phenomenon also occurs among young people aged 16 to 24, as approximately 69% of them alter the dosage of their medication. Furthermore, easy access to medication was a determining factor in self-medication among this group^(5)^.

From this perspective, the growing pursuit of the perfect body in contemporary society, coupled with aesthetic pressure and internalization of unrealistic standards, amplified by access to digital media, creates an environment conducive to body dissatisfaction among young people. Thus, the search for quick results significantly contributes to decisions that are harmful to health, such as the use of weight-loss drugs and anabolic steroids among adolescents^(6)^.

Another problem is the use of narcotic and psychoactive drugs among adolescents. The first experience with drugs and psychoactive substances frequently occurs in this age group. This highlights the importance of studying this phenomenon among young people, since, driven by curiosity and influence, these substances are consumed experimentally^(7)^.

The Brazilian National Committee for the Promotion of the Rational Use of Medications reinforces the importance and necessity of actions to curb abuses related to the use of medications. Among the strategic recommendations to achieve the proposed objective are encouraging discussion about the problem, combining efforts with other work already developed, and encouraging scientific production in related areas^(8)^. With this in mind, the present study aims to analyze the prevalence and factors associated with self-medication at some point in life and its current use among high school students in the Greater Vitória Metropolitan Region (GVMR), Espírito Santo.

## METHOD

### Study Design

This study has a cross-sectional design, carried out with data from a school-based study entitled *“Pesquisa estadual de saúde do escolar capixaba – PESC: uma análise dos escolares do ensino médio”*, conducted from March to December 2023, with the pilot study carried out in February of the same year.

### Context

The study was conducted in the GVMR, which comprises seven municipalities in the state of Espírito Santo, Brazil: Vitória, Vila Velha, Serra, Cariacica, Viana, Guarapari, and Fundão^(9)^. The estimated population of the GVMR for 2022 was 1,880,828 people, representing approximately 49% of the population of Espírito Santo^(10)^. There is no specific data on the adolescent population in the GVMR, but it is estimated that 63,255 students are enrolled in high school. This region has 171 high schools, both public and private^(11)^.

### Participants

The research audience included high school students from the GVMR. The sampling process was based on data from the 2020 *Instituto Nacional de Estudos e Pesquisas Educacionais Anísio Teixeira* to select schools, which were separated according to the proportion of students in each stratum: public and private school systems. For each stratum, an “N” was calculated using simple random sampling, corrected for complex samples at a later stage using the Statistical Package for the Social Sciences version 26.0, ensuring 95% confidence and a maximum error of 5%. For the municipal strata, the sample parameters were guaranteed to be 95% confidence and a maximum error of 10%, with a minimum sample size of 4,416.

### Data Collection

The questionnaire was answered directly by students using a mobile data collection device (tablet), which contained the structured questionnaire. This instrument was chosen to expedite the research process, as its familiar technology likely generates greater interest among adolescents in participating in data collection and is easier to use. It is important to note that only students with formal consent from their guardians, through the signing of the Informed Consent Form, and the assent of the adolescents themselves, through the Informed Assent Form, participated in the research. All participants were previously informed about the research objectives, as well as its potential risks and benefits.

### Outcome

The outcomes studied were self-medication at some point in life (yes/no) and current self-medication (yes/no). The outcomes were constructed from the use of weight-loss drugs (Ritalin, Anfepramone, Femproporex, Mazindol, Hipofagin, Inibex, Desobesi, Moderine, Absten, Fagolipo, and Dualid), anabolic steroids (Anabolex, Androlone, Androviron, Decadurabolin, Durabolin, Sustanon, Equipoise, and Parabolan), tranquilizers (Diazepam, Dienpax, Valium, Lorax, Rohypnol, Psicosedin, Somalium, Apraz, Rivotril, Dormonid, Bromazepam, Frontal, Olcadil, and Zolpidem), or narcotics (Morphine, Ketamine, Tylex, Setux, Sylador, Tramal, Dolantina, Fentanyl and Belacodid).

### Independents

Independent variables are composed of sex (female/male), age group (14 to 15, 16 to 17 or 18 to 19 years), gender identity (cisgender: yes or no), sexual orientation (heterosexual or LGBT+), race/skin color (white, black or brown), marital status (with or without partner), socioeconomic classification (A, B1, B2, C1, C2 or C3, D or E), type of school (public or private), whether they currently have any work, employment or business (no or yes), religion (none, Catholic, Evangelical or other) and marital status of parents (live together: yes or no).

### Data Analysis

Data were initially presented using absolute frequency, relative frequency, and 95% Confidence Interval (95%CI). Subsequently, data were assessed through bivariate analysis, using Pearson’s chi-square test to establish possible relationships, and through unadjusted and adjusted multivariate analysis, using the Prevalence Ratio (PR), with robust variance by Poisson Regression, considering for entry into the adjustment variables with statistical significance at 0.20 and retaining those with a p-value <0.05. Data analyses were conducted using the statistical software Stata 17.0.

This research was submitted to the *Universidade Federal do Espírito Santo* Research Ethics Committee and received approval through Opinion 5,900,370, guaranteeing compliance with all necessary ethical requirements.

## RESULTS

It is observed that the prevalence of self-medication at some point in life was 22.7% (95%CI: 21.5–23.9) in the overall sample. Female students (28.5%; 95%CI: 26.8–30.3) showed higher frequencies compared to male students (15.7%; 95%CI: 14.2–17.3). Furthermore, 7.7% (95%CI: 6.9–8.5) of participants reported current self-medication, which was also more prevalent among girls (10.3%; 95%CI: 9.2–11.6) compared to boys (4.5%; 95%CI: 3.7–5.4) (Table 1).

**Table 1:** Self-medication at some point in life and current self-medication among high school students in the Greater Vitória Metropolitan Region – Espírito Santo, Brazil, March to December, 2023 (N = 4.613).

Variables	General sample			Female			Male		
	N	%	95%CI	N	%	95%CI	N	%	95%CI
Has ever self-medicated									
No	3567	77.3	76.1–78.5	1790	71.5	69.7–73.2	1777	84.3	82.7–85.8
Yes	1046	22.7	21.5–23.9	715	28.5	26.8–30.3	330	15.7	14.2–17.3
Current self-medication									
No	4260	92.4	91.5–93.1	2247	89.7	88.4–90.8	2013	95.5	94.6–96.3
Yes	353	7.7	6.9–8.5	258	10.3	9.2–11.6	94	4.5	3.7–5.4

*One missing result in the male sex; N – absolute frequency; % – relative frequency; 95%CI – 95% Confidence Interval.

Table 2 shows that self-medication at some point in life and current self-medication were related to the variables sex, gender identity, sexual orientation, marital status, economic class, and religion. The variable parental marital status only showed a relationship with self-medication at some point in life, while working and having some kind of job, employment, or business were related to current self-medication (p < 0.05).

**Table 2:** Distribution of lifetime self-medication and current self-medication among high school students in the Greater Vitória Metropolitan Region – Espírito Santo, Brazil, March to December, 2023 (N = 4.613).

Variables	Has ever self–medicated				Current self–medication			
	N	%	95%CI	p–value	N	%	95%CI	p–value
**Sex**				<0,001				<0,001
Female	715	28.5	26.8–30.3		258	10.3	9.2–11.6	
Male	330	15.7	14.2–17.3		94	4.5	3.7–5.4	
**Age range**				0.168				0.405
14 to 15	213	22.0	19.5–24.7		84	8.7	7.0–10.6	
16 to 17	678	22.3	20.9–21.8		223	7.3	6.5–8.3	
18 to 19	155	25.7	22.3–29.3		46	7.6	5.8–10.0	
**Cisgender**				<0.001				0.001
Yes	948	22.0	20.7–23.2		315	7.3	6.6–8.1	
No	96	32.9	27.8–38.5		37	12.7	9.3–17.7	
**Sexual orientation**				<0.001				<0.001
Heterosexual	700	19.1	17.9–20.4		215	5.9	5.2–6.7	
LGBT+	345	36.4	33.4–39.5		137	14.4	12.3–16.8	
**Race/skin color**				0.726				0.098
White	387	21.8	19.9–23.8		125	7.0	5.9–8.3	
Black	173	22.8	19.9–25.9		71	9.3	7.5–11.6	
Brown	445	22.8	21.0–24.8		139	7.1	6.1–8.4	
**Marital status**				<0.001				0.045
With a partner	324	26.4	24.0–28.9		243	7.2	6.4–8.1	
Without a partner	722	21.3	20.0–28.9		110	9.0	7.5–10.7	
**Socioeconomic classification**				0.001				0.001
A	212	27.8	24.7–31.2		212	27.8	24.7–31.1	
B1/B2	465	21.4	19.7–23.1		465	21.4	19.8–23.1	
C1/C2/D/E	367	22.0	20.1–24.1		367	22.0	20.1–24.1	
**Type of school**				0.087				0.140
Public	678	21.9	20.5–23.4		224	7.3	6.4–8.2	
Private	368	24.2	22.1–26.4		129	8.5	7.2–10.0	
**Has currently a job, employment, or business**				0.054				0.012
No	769	22.0	20.7–23.4		248	7.11	6.3–8.0	
Yes	277	24.8	22.3–27.4		105	9.4	7.8–11.2	
**Religion**				<0.001				<0.001
None	268	24.6	22.1–27.3		95	8.7	7.2–10.6	
Catholic	242	20.5	18.3–22.9		79	6.7	5.4–8.3	
Evangelical	406	20.2	18.5–22.0		132	6.6	5.6–7.7	
Other	130	38.5	33.4–43.8		47	13.9	10.7–18.0	
**Parents’ marital status – they live together**				0.036				0.455
Yes	517	21.4	19.8–23.1		178	7.4	6.4–8.5	
No	519	24.0	22.2–25.8		172	8.0	6.9–9.2	

N – absolute frequency; % – relative frequency; 95%CI – 95% Confidence Interval.

After adjusting for confounding factors, it was found that female students had a 67% higher prevalence of self-medication at some point in their lives compared to male students (aPR= 1.67; 95%CI: 1.48 – 1.89; p < 0.001). Furthermore, a higher frequency was observed in the 18-19 year age group (aPR= 1.24; 95%CI: 1.04–1.48; p = 0.044) when compared to the 14–15 year age group. Similarly, LGBTQIA+ participants (aPR: 1.55), those with higher socioeconomic status (aPR: 1.36), those who declared following religions other than Evangelical and Catholic (aPR: 1.50), and those who had work, employment, or a business (aPR: 1.15) showed higher prevalences of self-medication at some point in their lives (Table 3).

**Table 3:** Unadjusted and adjusted prevalence ratio of lifetime self-medication among high school students in the Greater Vitória Metropolitan Region – Espírito Santo, Brazil, March to December, 2023.

Variables	Has ever self–medicated					
	Unadjusted PR	95%CI	p–value	Adjusted PR	95%CI	p–value
**Sex**			<0.001			<0.001
Female	1.82	1.62–2.05		1.67	1.48–1.89	
Male	1.0			1.0		
**Age range**			0.159			0.044
14 to 15	1.0			1.0		
16 to 17	1.02	0.89–1.16		1.05	0.92–1.20	
18 to 19	1.17	0.98–1.40		1.24	1.04–1.48	
**Cisgender**			<0.001			0.173
Yes	1.0			1.0		
No	1.50	1.26–1.78		1.13	0.95–1.35	
**Sexual orientation**			<0.001			<0.001
Heterosexual	1.0			1.0		
LGBT+	1.90	1.71–2.12		1.55	1.37–1.74	
**Race/skin color**			0.726			–
White	1.0			–	–	
Black	1.04	0.89–1.22		–	–	
Brown	1.04	0.93–1.18		–	–	
**Marital status**			<0.001			0.159
With a partner	1.24	1.10–1.39		1.09	0.97–1.74	
Without a partner	1.0			1.0		
**Socioeconomic classification**			0.001			<0.001
A	1.26	1.09–1.46		1.36	1.18–1.57	
B	0.97	0.86–1.09		1.01	0.90–1.14	
C//D/E						
**Type of school**			0.086			0.338
Public	1.0			1.0		
Private	1.10	0.99–1.23		1.06	0.94–1.21	
**Has currently a job, employment, or business**			0.052			0.018
No	1.0			1.0		
Yes	1.13	1.00–1.27		1.15	1.02–1.29	
**Religion**			<0.001			<0.001
None	1.0			1.0		
Catholic	0.83	0.72–0.97		0.95	0.81–1.11	
Evangelical	0.82	0.72–0.94		0.96	0.83–1.10	
Other	1.56	1.32–1.85		1.50	1.27–1.77	
**Parents’ marital status – they live together**			0.036			0.241
Yes	1.0			1.0		
No	1.12	1.01–1.25		1.07	0.96–1.91	

PR – Prevalence Ratio; 95%CI – 95% Confidence Interval.

In adjusted analysis, the prevalence of current self-medication in females was approximately twice as high (aPR = 2.12; 95%CI: 1.65–2.72; p < 0.001) compared to males. LGBTQIA+ students also showed higher frequencies (aPR = 2.04; 95%CI: 1.64–2.54; p < 0.001) compared to heterosexuals. Regarding race/skin color, self-declared black adolescents showed higher prevalences of current self-medication (aPR: 1.49), as well as this practice being twice as high among students from economic class “A” (aPR = 2.27; 95%CI: 1.73–2.97; p < 0.001) and more frequently observed among those who had a job (aPR = 1.39; 95%CI: 1.12–1.74; p = 0.003) (Table 4).

**Table 4:** Unadjusted and adjusted prevalence ratio of current self-medication among high school students in the Greater Vitória Metropolitan Region – Espírito Santo, Brazil, March to December, 2023.

Variables	Current self-medication					
	Unadjusted PR	95%CI	p-value	Adjusted PR	95%CI	p-value
**Sex**			<0.001			<0.001
Female	2.31	1.84–2.90		2.12	1.65–2.72	
Male	1.0			1.0		
**Age range**			0.404			–
14 to 15	1.0			–	–	
16 to 17	0.85	0.67–1.08		–	–	
18 to 19	0.88	0.63–1.24		–	–	
**Cisgender**			0.001			0.594
Yes	1.0			1.0		
No	1.74	1.26–2.39		1.10	0.78–1.56	
**Sexual orientation**			<0.001			<0.001
Heterosexual	1.0			1.0		
LGBT+	2.50	2.01–3.01		2.04	1.64–2.54	
**Race/skin color**			0.097			0.019
White	1.0			1.0		
Black	1.33	1.00–1.75		1.49	1.13–1.96	
Brown	1.01	0.80–1.28		1.13	0.90–1.42	
**Marital status**			0.044			0.930
With a partner	1.25	1.01–1.55		0.99	0.79–1.25	
Without a partner	1.0			1.0		
**Socioeconomic classification**			<0.001			<0.001
A	1.85	1.42–2.40		2.27	1.73–2.97	
B	1.04	0.82–1.32		1.20	0.94–1.54	
C//D/E	1.0			1.0		
**Type of school**			0.140			0.649
Public	1.0			1.0		
Private	1.20	0.95–1.44		1.06	0.82–1.37	
**Has currently a job, employment, or business**			0.012			0.003
No	1.0			1.0		
Yes	1.32	1.06–1.65		1.39	1.12–1.74	
**Religion**			<0.001			0.129
None	1.0			1.0		
Catholic	0.77	0.58–1.02		0.90	0.66–1.22	
Evangelical	0.75	0.58–0.97		0.90	0.68–1.18	
Other	1.59	1.15–2.21		1.32	0.94–1.86	
**Parents’ marital status – they live together**			0.455			–
Yes	1.0			–	–	
No	1.08	0.88–1.32		–	–	

PR – Prevalence Ratio; 95%CI – 95% Confidence Interval.

## DISCUSSION

This study shows a significant number of adolescents who reported self-medication at some point in their lives and current self-medication. This result supports national findings that reinforce the high prevalence of medication use among young people. A cross-sectional study conducted in Porto Alegre indicated that approximately half of the students reported using medication in the seven days prior to data collection. Part of this consumption may be associated with self-medication practice, which is facilitated by easy access to nonprescribed medications, purchased directly from pharmacies or found stored at home. Adding to this problem is exposure to harmful campaigns that promote and encourage consumption even in the absence of professional guidance, targeting individuals who may not have the critical thinking skills to recognize the limits of medication use^(12)^.

Another factor justifying this behavior among schoolchildren is the limited knowledge about the rational use of medication. Poor understanding of drug indications, dosages, and application has been identified as a determinant of risky behaviors, such as polypharmacy, taking excessive doses of medication, and not reading the package insert. An international study reinforced this understanding by demonstrating that adolescents with low levels of knowledge about medications showed a higher prevalence of self-medication^(13)^. Furthermore, how adolescents perceive their own health directly influences their care practices. Adolescents who have a poor view of their health tend to use more medications from different therapeutic classes, reinforcing the association between negative health perception and increased medication consumption^(14)^.

Friendship networks have emerged as one of the primary means of accessing non-prescription tranquilizers and sedatives among adolescents^(15)^. The search for sensations and recreational effects was associated with the acquisition of these medications through friends, this practice of peer-sharing being common, especially among young people who have already had legitimate prescriptions. Thus, the vulnerability evidenced by the lack of knowledge about the risks of uncontrolled use of these drugs highlights the seriousness of high-risk substance use among schoolchildren^(16)^.

The highest prevalence of self-medication, whether at some point in life or currently, was observed in female adolescents. A cohort study conducted in Pelotas found that women use significantly more medication than men, even when contraceptives are excluded from the analysis^(17)^. Similarly, international standards reaffirm this gender difference. An international cross-sectional analysis conducted in Spain with students between 2016 and 2021 observed that the non-prescribed use of tranquilizers, such as benzodiazepines and hypnotics, among girls was about twice as high compared to boys. Furthermore, the variables independently and significantly associated with this higher probability were alcohol and cannabis use in the last 30 days^(18)^.

In this regard, incorporating a new gender perspective on the use of psychoactive substances allows for a broader understanding of the different consumption patterns between men and women. Among the causes are the higher incidence of depressive and anxiety disorders among women, even in adolescence, and their unequal position compared to men. It is also considered that this population tends to perceive and report their symptoms more frequently, leading them to seek healthcare services more often and contributing to a process of medicalization of their emotional experiences. Subsequently, they use these medications with altered dosages^(18)^.

Another finding of this research is the higher prevalence of self-medication at some point in life and currently among adolescents from socioeconomic class A. These results support a national cross-sectional study, conducted in the 27 Brazilian capitals with high school students from public and private schools, highlighting the non-prescribed use of tranquilizers/sedatives, which is more common among those from higher socioeconomic classes^(15)^. One possible hypothesis to explain this finding is that individuals in more favorable economic situations have greater access to healthcare services and, consequently, greater access to these medications, which are often obtained in their own homes, increasing misuse among family members^(19)^.

Substance use among LGBT+ adolescents is still underexplored in the literature, although it represents an important finding for understanding public health issues involving this population, such as social vulnerability and the psychosocial challenges they face. The current study indicated that the lifetime prevalence of self-medication was almost twice as high among LGBT+ students compared to heterosexuals. This relationship held true for current self-medication, becoming 2.5 times more prevalent among them. These results are consistent with an analysis of data from the United States that demonstrated that adolescents from sexual minorities, regardless of sex, were more likely to report moderate or severe misuse of anabolic-androgenic steroids (AAS) compared to their heterosexual peers^(20)^.

In this context, one factor to consider is minority stress theory, which explains this type of behavior as a response to the impacts of social stigma, rejection, and discrimination. In this context, substance use can be a coping mechanism for the conflicts experienced by these adolescents^(21)^. Another international study reinforces these findings, pointing out that male adolescents belonging to sexual minorities showed a significantly higher prevalence of AAS misuse throughout their lives, attributing this behavior to the effects of minority stress and greater body dissatisfaction, factors that can act as motivators and as a way of coping and seeking conformity to aesthetic standards or gender identity^(22)^.

Analyzing the current study, this research found that adolescents with some form of employment had a higher prevalence of self-medication. A study conducted in the United States using data from the Brazilian National Surveys of Health and Human Development found an association between being employed and increased rates of recent and excessive substance use among adolescents. This phenomenon can be explained by several factors, such as students entering the job market, which exposes them to other social circles with people of different ages, favoring the use of illicit substances. Access to these substances is facilitated by increased income from work. Another factor mentioned is the stress and overload of balancing studies with work, which can lead adolescents to seek self-medication as an inappropriate way to cope with symptoms such as fatigue, difficulty sleeping, and anxiety^(23)^.

This study has some limitations. The first refers to the cross-sectional nature of the study, which makes it difficult to establish causal relationships, since it is not possible to determine the sequence between exposure and outcome. Another limitation is the possibility of recall bias in data collection. Data collection was based on self-reporting, which may have caused participants to forget the name or class of medications. Furthermore, information bias is considered, which may have led to data omission, either due to forgetfulness or reluctance to disclose socially unacceptable practices. Despite this, the self-administered questionnaire minimizes reluctance to disclose socially unacceptable practices.

## CONCLUSION

The data presented in the study revealed a significant prevalence of self-medication at some point in life and current self-medication among high school students in the GVMR. Higher frequencies were observed in the variables female sex, non-cisgender students, LGBTQIA+, higher socioeconomic classification, those who had some kind of employment, and those who reported religions other than Catholic and Evangelical. Thus, the findings reinforce the complexity of self-medication among adolescents, which involves not only issues of access and availability of medications, but also psychosocial and cultural aspects. Furthermore, they highlight how the home and social environment are determining factors in this process of experimentation and inappropriate use of substances among students. Therefore, the results of this research show the need for intersectoral strategies that carry out health education actions and promote public policies aimed at promoting the rational use of medications among students.

## Data Availability

The entire dataset supporting the results of this study was published in the article itself.
