# Supplementary material for: Prevalence and factors associated with self-medication among schoolchildren in the Metropolitan Region of Vitória, Brazil
Source: Rev Esc Enferm USP. 2026 Mar 16;60:e20250395. doi: 10.1590/1980-220X-REEUSP-2025-0395en (PMC13037791; doi:10.1590/1980-220X-REEUSP-2025-0395en)
Supplement: Translation

# Translation: Prevalência e fatores associados à automedicação entre escolares na Região Metropolitana de Vitória, Brasil

**DOI:** 10.1590/1980-220X-REEUSP-2025-0395pt

**Authors:** Kamila Rocha Mairink, Fernanda Garcia Gabira Miguez, Carlos Augusto Lopes, Franciele Foschiera Camboin, Nathalia Miguel Teixeira Santana, Nathalia Borba Raposo Pereira, Franciéle Marabotti Costa Leite

**Affiliations:** 1 Universidade Federal do Espírito Santo, Departamento de Enfermagem, Vitória, ES, Brasil.; 2 Universidade Federal do Espírito Santo, Programa de Pós-Graduação em Saúde Coletiva, Vitória, ES, Brasil.; 3 Universidade Federal do Espírito Santo, Programa de Ciências Sociais, Vitória, ES, Brasil.; 4 Universidade Estadual do Oeste do Paraná, Cascavel, PR, Brasil.; 5 Universidade Federal do Espírito Santo, Programa de Pós-Graduação em Psicologia, Vitória, ES, Brasil.

**Keywords:** Adolescentes, Saúde do Adolescente, Automedicação, Medicamentos sem Prescrição

## Abstract

**Objetivo:**

Analisar a prevalência e os fatores associados à automedicação alguma vez na vida e seu uso atual entre escolares do ensino médio da Região Metropolitana da Grande Vitória, Espírito Santo.

**Método:**

Estudo transversal, de base escolar, realizado entre março e dezembro de 2023, com 4.416 escolares. A experimentação e o uso atual de medicamentos sem prescrição médica foram o desfecho. As variáveis independentes categóricas foram sexo, faixa etária, identidade de gênero, raça/cor da pele, classificação socioeconômica, tipo de escola, possuir algum trabalho, emprego ou negócio atualmente, religião e situação conjugal dos pais. Os dados foram avaliados por teste qui-quadrado de Pearson e Regressão de Poisson.

**Resultados:**

A prevalência da automedicação alguma vez na vida entre os escolares foi de 22,7% (IC95%: 21,5–23,9), sendo mais prevalente no sexo feminino (28,5%; IC95%: 26,8–30,3). Há maior prevalência da automedicação alguma vez na vida e automedicação atual entre estudantes do sexo feminino, LGBT+, de classe socioeconômica elevada e com vínculo de trabalho.

**Conclusão:**

Os achados reforçam a complexibilidade da automedicação entre os adolescentes, que envolve não apenas questões de acesso e disponibilidade de medicamentos, mas também aspectos psicossociais e culturais.

## INTRODUÇÃO

O acesso aos medicamentos proporcionou à sociedade benefícios que envolvem a prevenção, manutenção e recuperação da saúde, contribuindo, de forma geral, para prolongar a vitalidade humana^(1)^. No entanto, apesar desses avanços inegáveis, o uso irracional de medicamentos se configura como um problema de saúde pública, uma vez que sua utilização inadequada resulta em riscos para a saúde^(2)^.

De acordo com a Organização Mundial da Saúde, mais da metade de todos os medicamentos são dispensados, vendidos e prescritos incorretamente. Além disso, acima de 50% de todos os países não realizam políticas básicas para promover o uso racional deles^(3)^. No Brasil, no entanto, a utilização incorreta de medicamentos está relacionada à prescrição não orientada por diretrizes técnicas e clínicas estabelecidas por órgãos reguladores e sociedades científicas, à automedicação inapropriada e à ampla disponibilidade de medicamentos para compra no mercado^(4)^.

Segundo levantamento feito pelo Conselho Federal de Farmácia, no ano de 2019, 77% dos brasileiros consumiram medicamentos por conta própria entre julho e dezembro. Esse fenômeno também ocorre entre os jovens com idade de 16 a 24 anos, pois cerca de 69% deles alteram a posologia do medicamento. Além disso, a facilidade de acesso ao remédio foi um fator determinante para a automedicação entre esse grupo^(5)^.

Nessa perspectiva, a crescente busca pelo corpo perfeito na sociedade contemporânea, associada à pressão estética e à internalização de padrões irreais, potencializada pelo acesso às mídias digitais, cria um ambiente propício à insatisfação corporal entre os jovens. Assim, a busca por resultados rápidos contribui significantemente para tomada de decisões lesivas à saúde, como o uso de medicamentos emagrecedores e anabolizantes entre adolescentes^(6)^.

Outra problemática é o uso de medicamentos entorpecentes e psicoativos entre os adolescentes. A primeira experiência com drogas e substâncias psicoativas ocorre frequentemente nessa faixa etária. Isso evidencia a importância de estudar esse fenômeno entre os jovens, visto que, movidos pela curiosidade e influência, essas substâncias são consumidas de forma experimental^(7)^.

O Comitê Nacional para a Promoção do Uso Racional de Medicamentos reforça a importância e a necessidade de ações para conter os abusos relacionados ao uso de medicamentos. Entre as recomendações estratégicas para chegar ao objetivo proposto, estão o incentivo à discussão sobre a problemática, a soma dos esforços com outros trabalhos já desenvolvidos e o estímulo à produção científica nas áreas relacionadas^(8)^. Com esse intuito, o presente estudo tem como objetivo analisar a prevalência e os fatores associados à automedicação alguma vez na vida e seu uso atual entre escolares do ensino médio da Região Metropolitana da Grande Vitória (RMGV), Espírito Santo.

## MÉTODO

### Delineamento do Estudo

Este estudo possui delineamento transversal, realizado com dados de estudo de base escolar intitulado “Pesquisa estadual de saúde do escolar capixaba – PESC: uma análise dos escolares do ensino médio”, conduzido de março a dezembro de 2023, sendo o estudo piloto realizado em fevereiro do mesmo ano.

### Contexto

A realização do estudo se deu na RMGV, composta por sete municípios do estado do Espírito Santo, Brasil, sendo eles Vitória, Vila Velha, Serra, Cariacica, Viana, Guarapari e Fundão^(9)^. A população estimada na RMGV para o ano de 2022 era de 1.880.828 pessoas, e isso representa cerca de 49% da população capixaba^(10)^. Não há dados específicos sobre a população de adolescentes na RMGV, mas estima-se que 63.255 alunos estão matriculados no ensino médio. Essa região possui 171 escolas de ensino médio, entre públicas e privadas^(11)^.

### Participantes

O público da pesquisa incluiu estudantes do ensino médio da RMGV. O processo de amostragem foi baseado nos dados do Instituto Nacional de Estudos e Pesquisas Educacionais Anísio Teixeira de 2020 para a seleção das escolas, que foram separadas de acordo com a proporção de estudantes em cada estrato: rede pública e rede privada. Para cada estrato, foram calculados um “N” usando amostragem aleatória simples, corrigidas para amostras complexas em um segundo momento usando o programa estatístico *Statistical Package for the Social Sciences*, 26.0, garantindo 95% de confiança e erro máximo de 5%. Para os estratos de município, foram garantidos como paramentos da amostra 95% de confiança e erro máximo de 10%, sendo a amostra mínima de 4.416.

### Coleta de Dados

O questionário foi respondido diretamente pelo aluno por meio do Dispositivo Móvel de Coleta (*tablet*), no qual estava inserido o questionário estruturado. A escolha desse instrumento teve o objetivo de agilizar o processo de execução da pesquisa, visto que, por ser uma tecnologia conhecida, desperte maior interesse dos adolescentes em participar da coleta de dados, além de menor dificuldade no manuseio. Vale destacar que somente participaram da pesquisa os estudantes com o consentimento formal dos responsáveis, por meio da assinatura do Termo de Consentimento Livre e Esclarecido, e o assentimento dos próprios adolescentes, por meio do Termo de Assentimento Livre e Esclarecido. Todos os participantes foram previamente informados sobre os objetivos da pesquisa, bem como seus possíveis riscos e benefícios.

### Desfecho

Os desfechos em estudo foram a automedicação alguma vez na vida (sim/não) e automedicação atual (sim/não). Os desfechos foram construídos a partir do uso de remédios para emagrecer (Ritalina, Anfepramona, Femproporex, Mazindol, Hipofagin, Inibex, Desobesi, Moderine, Absten, Fagolipo e Dualid), anabolizantes (Anabolex, Androlone, Androviron, Decadurabolin, Durabolin, Durateston, Equipoise e Parabolan), tranquilizantes (Diazepam, Dienpax, Valium, Lorax, Rohypnol, Psicosedin, Somalium, Apraz, Rivotril, Dormonid, Bromazepam, Frontal, Olcadil e Zolpidem), ou entorpecentes (Morfina, Cetamina, Tylex, Setux, Sylador, Tramal, Dolantina, Fentanil e Belacodid).

### Independentes

As variáveis independentes são compostas por sexo (feminino/masculino), faixa etária (14 a 15, 16 a 17 ou 18 a 19 anos), identidade de gênero (cisgênero: sim ou não), orientação sexual (heterossexual ou LGBT+), raça/cor da pele (branca, preta ou parda), estado civil (com ou sem companheiro), classificação socioeconômica (A, B1, B2, C1, C2 ou C3, D ou E), tipo de escola (pública ou privada), se atualmente possui algum trabalho, emprego ou negócio (não ou sim), religião (nenhuma, católica, evangélica ou outras) e situação conjugal dos pais (vivem juntos: sim ou não).

### Análise de Dados

Os dados foram primeiramente apresentados mediante frequência absoluta, frequência relativa e Intervalo de Confiança de 95% (IC95%). Posteriormente, os dados foram avaliados através de análise bivariada, com uso do teste qui-quadrado de Pearson para estabelecer possíveis relações, e de análise multivariada bruta e ajustada, através da Razão de Prevalência (RP), com variância robusta por Regressão de Poisson, sendo consideradas para entrada no ajuste as variáveis com significância estatística em 0,20 e mantidas aquelas com valor de p < 0,05. As análises de dados foram conduzidas no programa estatístico Stata 17.0.

Esta pesquisa foi submetida ao Comitê de Ética em Pesquisa da Universidade Federal do Espírito Santo, e recebeu aprovação por meio do Parecer n° 5.900.370, garantindo o cumprimento de todas as exigências éticas necessárias.

## RESULTADOS

Observa-se que a prevalência da automedicação alguma vez na vida foi de 22,7% (IC95%: 21,5–23,9) na amostra geral. As estudantes do sexo feminino (28,5%; IC95%: 26,8–30,3) apresentaram maiores frequências em comparação aos do sexo masculino (15,7%; IC95%: 14,2–17,3). Além disso, 7,7% (IC95%: 6,9–8,5) dos participantes referiram a automedicação atual, sendo também mais prevalente entre as meninas (10,3%; IC95%: 9,2–11,6) em relação aos meninos (4,5%; IC95%: 3,7–5,4) (Tabela 1).

**Tabela 1:** Automedicação alguma vez na vida e automedicação atual entre os escolares do ensino médio da Região Metropolitana da Grande Vitória – Espírito Santo, Brasil, março a dezembro, 2023 (N = 4.613).

Variáveis	Amostra geral			Feminino			Masculino		
	N	%	IC95%	N	%	IC95%	N	%	IC95%
Automedicação alguma vez na vida									
Não	3567	77,3	76,1–78,5	1790	71,5	69,7–73,2	1777	84,3	82,7–85,8
Sim	1046	22,7	21,5–23,9	715	28,5	26,8–30,3	330	15,7	14,2–17,3
Automedicação atual									
Não	4260	92,4	91,5–93,1	2247	89,7	88,4–90,8	2013	95,5	94,6–96,3
Sim	353	7,7	6,9–8,5	258	10,3	9,2–11,6	94	4,5	3,7–5,4

*Un *missing* no sexo masculino; N – frequência absoluta; % – frequência relativa; IC95% – Intervalo de Confiança de 95%.

Nota-se, na Tabela 2, que a automedicação alguma vez na vida e a automedicação atual estiveram relacionadas às variáveis sexo, identidade de gênero, orientação sexual, estado civil, classe econômica e religião. A variável situação conjugal dos pais somente apresentou relação com a automedicação alguma vez na vida, enquanto trabalhar e possuir algum trabalho, emprego ou negócio estiveram relacionados à automedicação atual (p < 0,05).

**Tabela 2:** Distribuição da automedicação alguma vez na vida e automedicação atual entre os escolares do ensino médio da Região Metropolitana da Grande Vitória – Espírito Santo, Brasil, março a dezembro, 2023 (N = 4.613).

Variáveis	Automedicação alguma vez na vida				Automedicação atual			
	N	%	IC95%	Valor de p	N	%	IC95%	Valor de p
**Sexo**				<0,001				<0,001
Feminino	715	28,5	26,8–30,3		258	10,3	9,2–11,6	
Masculino	330	15,7	14,2–17,3		94	4,5	3,7–5,4	
**Faixa etária**				0,168				0,405
14 a 15	213	22,0	19,5–24,7		84	8,7	7,0–10,6	
16 a 17	678	22,3	20,9–21,8		223	7,3	6,5–8,3	
18 a 19	155	25,7	22,3–29,3		46	7,6	5,8–10,0	
**Cisgênero**				<0,001				0,001
Sim	948	22,0	20,7–23,2		315	7,3	6,6–8,1	
Não	96	32,9	27,8–38,5		37	12,7	9,3–17,7	
**Orientação sexual**				<0,001				<0,001
Heterossexual	700	19,1	17,9–20,4		215	5,9	5,2–6,7	
LGBT+	345	36,4	33,4–39,5		137	14,4	12,3–16,8	
**Raça/cor da pele**				0,726				0,098
Branca	387	21,8	19,9–23,8		125	7,0	5,9–8,3	
Preta	173	22,8	19,9–25,9		71	9,3	7,5–11,6	
Parda	445	22,8	21,0–24,8		139	7,1	6,1–8,4	
**Estado civil**				<0,001				0,045
Com companheiro	324	26,4	24,0–28,9		243	7,2	6,4–8,1	
Sem companheiro	722	21,3	20,0–28,9		110	9,0	7,5–10,7	
**Classificação socioeconômica**				0,001				0,001
A	212	27,8	24,7–31,2		212	27,8	24,7–31,1	
B1/B2	465	21,4	19,7–23,1		465	21,4	19,8–23,1	
C1/C2/D/E	367	22,0	20,1–24,1		367	22,0	20,1–24,1	
**Tipo de escola**				0,087				0,140
Pública	678	21,9	20,5–23,4		224	7,3	6,4–8,2	
Privada	368	24,2	22,1–26,4		129	8,5	7,2–10,0	
**Atualmente possui algum trabalho, emprego ou negócio**				0,054				0,012
Não	769	22,0	20,7–23,4		248	7,11	6,3–8,0	
Sim	277	24,8	22,3–27,4		105	9,4	7,8–11,2	
**Religião**				<0,001				<0,001
Nenhuma	268	24,6	22,1–27,3		95	8,7	7,2–10,6	
Católica	242	20,5	18,3–22,9		79	6,7	5,4–8,3	
Evangélica	406	20,2	18,5–22,0		132	6,6	5,6–7,7	
Outras	130	38,5	33,4–43,8		47	13,9	10,7–18,0	
**Situação conjugal dos pais – vivem juntos**				0,036				0,455
Sim	517	21,4	19,8–23,1		178	7,4	6,4–8,5	
Não	519	24,0	22,2–25,8		172	8,0	6,9–9,2	

N – frequência absoluta; % – frequência relativa; IC95% – Intervalo de Confiança de 95%.

Após os ajustes para os fatores de confusão, verifica-se que estudantes do sexo feminino apresentaram uma prevalência 67% maior de automedicação alguma vez na vida em comparação aos do sexo masculino (RPa = 1,67; IC95%: 1,48–1,89; p < 0,001). Além disso, foi observada maior frequência na faixa etária de 18 a 19 anos (RPa = 1,24; IC95%: 1,04–1,48; p = 0,044) quando comparada ao grupo de 14 e 15 anos. De igual modo, participantes LGBTQIA+ (RPa: 1,55), de classificação socioeconômica mais elevada (RPa: 1,36), que declararam seguir outras religiões que não evangélicas e católicas (RPa: 1,50), e aqueles que possuíam trabalho, emprego ou negócio (RPa: 1,15) apresentaram maiores prevalências de automedicação alguma vez na vida (Tabela 3).

**Tabela 3:** Razão de prevalência bruta e ajustada da automedicação alguma vez na vida entre os escolares do ensino médio da Região Metropolitana da Grande Vitória – Espírito Santo, Brasil, março a dezembro, 2023.

Variáveis	Automedicação alguma vez na vida					
	RP bruto	IC95%	Valor de p	RP ajustada	IC95%	Valor de p
**Sexo**			<0,001			<0,001
Feminino	1,82	1,62–2,05		1,67	1,48–1,89	
Masculino	1,0			1,0		
**Faixa etária**			0,159			0,044
14 a 15	1,0			1,0		
16 a 17	1,02	0,89–1,16		1,05	0,92–1,20	
18 a 19	1,17	0,98–1,40		1,24	1,04–1,48	
**Cisgênero**			<0,001			0,173
Sim	1,0			1,0		
Não	1,50	1,26–1,78		1,13	0,95–1,35	
**Orientação sexual**			<0,001			<0,001
Heterossexual	1,0			1,0		
LGBT+	1,90	1,71–2,12		1,55	1,37–1,74	
**Raça/cor da pele**			0,726			–
Branca	1,0			–	–	
Preta	1,04	0,89–1,22		–	–	
Parda	1,04	0,93–1,18		–	–	
**Estado civil**			<0,001			0,159
Com companheiro	1,24	1,10–1,39		1,09	0,97–1,74	
Sem companheiro	1,0			1,0		
**Classificação socioeconômica**			0,001			<0,001
A	1,26	1,09–1,46		1,36	1,18–1,57	
B	0,97	0,86–1,09		1,01	0,90–1,14	
C//D/E						
**Tipo de escola**			0,086			0,338
Pública	1,0			1,0		
Privada	1,10	0,99–1,23		1,06	0,94–1,21	
**Atualmente possui algum trabalho, emprego ou negócio**			0,052			0,018
Não	1,0			1,0		
Sim	1,13	1,00–1,27		1,15	1,02–1,29	
**Religião**			<0,001			<0,001
Nenhuma	1,0			1,0		
Católica	0,83	0,72–0,97		0,95	0,81–1,11	
Evangélica	0,82	0,72–0,94		0,96	0,83–1,10	
Outras	1,56	1,32–1,85		1,50	1,27–1,77	
**Situação conjugal dos pais – vivem juntos**			0,036			0,241
Sim	1,0			1,0		
Não	1,12	1,01–1,25		1,07	0,96–1,91	

RP – Razão de Prevalência; IC95% – Intervalo de Confiança de 95%.

Na análise ajustada, a prevalência automedicação atual no sexo feminino foi cerca de duas vezes maior (RPa = 2,12; IC95%: 1,65–2,72; p < 0,001) em relação ao masculino. Estudantes LGBTQIA + também apresentaram maiores frequências (RPa = 2,04; IC95%: 1,64–2,54; p < 0,001) em comparação aos heterossexuais. Em relação à raça/cor da pele, adolescentes autodeclarados pretos apresentaram maiores prevalências de automedicação atual (RPa: 1,49), assim como essa prática foi duas vezes maior entre os estudantes de classe econômica “A” (RPa = 2,27; IC95%: 1,73–2,97; p < 0,001) e mais frequentemente observada entre os que possuíam trabalho (RPa = 1,39; IC95%: 1,12–1,74; p = 0,003) (Tabela 4).

**Tabela 4:** Razão de prevalência bruta e ajustada da automedicação atual entre os escolares do ensino médio da Região Metropolitana da Grande Vitória – Espírito Santo, Brasil, março a dezembro, 2023.

Variáveis	Automedicação atual					
	RP bruto	IC95%	Valor de p	RP ajustada	IC95%	Valor de p
**Sexo**			<0,001			<0,001
Feminino	2,31	1,84–2,90		2,12	1,65–2,72	
Masculino	1,0			1,0		
**Faixa etária**			0,404			–
14 a 15	1,0			–	–	
16 a 17	0,85	0,67–1,08		–	–	
18 a 19	0,88	0,63–1,24		–	–	
**Cisgênero**			0,001			0,594
Sim	1,0			1,0		
Não	1,74	1,26–2,39		1,10	0,78–1,56	
**Orientação sexual**			<0,001			<0,001
Heterossexual	1,0			1,0		
LGBT+	2,50	2,01–3,01		2,04	1,64–2,54	
**Raça/cor da pele**			0,097			0,019
Branca	1,0			1,0		
Preta	1,33	1,00–1,75		1,49	1,13–1,96	
Parda	1,01	0,80–1,28		1,13	0,90–1,42	
**Estado civil**			0,044			0,930
Com companheiro	1,25	1,01–1,55		0,99	0,79–1,25	
Sem companheiro	1,0			1,0		
**Classificação socioeconômica**			<0,001			<0,001
A	1,85	1,42–2,40		2,27	1,73–2,97	
B	1,04	0,82–1,32		1,20	0,94–1,54	
C//D/E	1,0			1,0		
**Tipo de escola**			0,140			0,649
Pública	1,0			1,0		
Privada	1,20	0,95–1,44		1,06	0,82–1,37	
**Atualmente possui algum trabalho, emprego ou negócio**			0,012			0,003
Não	1,0			1,0		
Sim	1,32	1,06–1,65		1,39	1,12–1,74	
**Religião**			<0,001			0,129
Nenhuma	1,0			1,0		
Católica	0,77	0,58–1,02		0,90	0,66–1,22	
Evangélica	0,75	0,58–0,97		0,90	0,68–1,18	
Outras	1,59	1,15–2,21		1,32	0,94–1,86	
**Situação conjugal dos pais – vivem juntos**			0,455			–
Sim	1,0			–	–	
Não	1,08	0,88–1,32		–	–	

RP – Razão de Prevalência; IC95% – Intervalo de Confiança de 95%.

## DISCUSSÃO

O presente estudo mostra uma quantidade relevante de adolescentes que relataram a automedicação alguma vez na vida e a automedicação atual. Esse resultado corrobora os achados nacionais que reforçam a alta prevalência de consumo de remédios entre os jovens. Pesquisa transversal, realizada em Porto Alegre, apontou que aproximadamente metade dos estudantes relatou ter utilizado medicamentos nos sete dias anteriores à coleta de dados. Parte desse consumo pode ser associado à prática da automedicação, que é facilitada pelo fácil acesso a medicamentos sem prescrição médica, adquiridos diretamente em farmácias ou encontrados estocados em casa. Soma-se a essa problemática a exposição a campanhas que se tornam prejudiciais, pois promovem e estimulam o consumo mesmo na ausência de orientação profissional a indivíduos que podem não ter uma postura crítica a ponto de reconhecerem o limite do uso^(12)^.

Outro fator que justifica essa conduta entre escolares é a limitação do conhecimento sobre o uso racional da medicação. A baixa compreensão sobre indicações, dosagens e aplicação do fármaco tem sido apontada como um dos determinantes para comportamentos de riscos, como praticar polifarmácia, tomar medicamentos com dosagens excessivas e não ler a bula. Estudo internacional reforçou essa compreensão ao demonstrar que adolescentes com baixo nível de conhecimento sobre medicamentos apresentaram maior prevalência de automedicação^(13)^. Além disso, a forma como os adolescentes percebem sua própria saúde influencia diretamente suas práticas de cuidado. Adolescentes que apresentam uma visão ruim da sua saúde tendem a utilizar mais medicamentos de diferentes classes terapêuticas, o que reforça a associação entre percepção negativa da saúde e o maior consumo de medicamentos^(14)^.

O ciclo de amizade configurou-se como um dos principais meios de acesso a medicamentos tranquilizantes e sedativos não prescritos entre adolescentes^(15)^. A busca por sensações e efeitos recreativos esteve associada à aquisição desses medicamentos através de amigos, sendo essa prática de compartilhamento entre pares comum, especialmente entre jovens que já tiveram prescrições legítimas. Desse modo, a vulnerabilidade evidenciada pela ausência de conhecimento sobre os riscos do uso descontrolado desses fármacos evidencia a gravidade do uso de substâncias de alto risco entre escolares^(16)^.

As maiores prevalências de automedicação, sejam alguma vez na vida ou atualmente, foram observadas em adolescentes do sexo feminino. Estudo de coorte realizado em Pelotas constatou que as mulheres usam mais medicamentos do que os homens significantemente, mesmo quando os anticoncepcionais são excluídos das análises^(17)^. De forma semelhante, padrões internacionais reafirmam essa diferença entre os gêneros. Análise internacional do tipo transversal, conduzida na Espanha com estudantes, entre 2016 e 2021, observou que o uso não prescrito de tranquilizantes, como benzodiazepínicos e hipnóticos, entre meninas era cerca de duas vezes maior quando comparado aos meninos. Além disso, as variáveis associadas de forma independente e significativa a essa maior probabilidade foram o uso de álcool e *cannabis* nos últimos 30 dias^(18)^.

Nesse sentido, a incorporação de uma nova perspectiva de gênero no uso de substâncias psicoativas permite ampliar a compreensão dos diferentes padrões de consumo entre homens e mulheres. Entre as causas, estão a maior incidência de quadros depressivos e ansiosos entre as mulheres, mesmo na adolescência, e sua posição desigual em relação aos homens. Considera-se também que essa população tende a perceber e relatar seus sintomas com maior frequência, o que a leva a procurar mais os serviços de saúde e a contribuir para um processo de medicalização de suas experiências emocionais. Posteriormente, elas utilizam esses medicamentos com alteração na posologia^(18)^.

Outro achado na presente pesquisa são as maiores prevalências da automedicação alguma vez na vida e atualmente entre os adolescentes de classe econômica A. Esses resultados corroboram pesquisa nacional do tipo transversal, realizada nas 27 capitais brasileiras com escolares do ensino médio de escolas públicas e privadas, evidenciando o uso não prescrito de tranquilizantes/sedativos e sendo mais comum entre aqueles com classes sociais mais altas^(15)^. Uma possível hipótese para explicar esse achado é que indivíduos em situações econômicas mais favoráveis têm mais acesso aos serviços de saúde e, consequentemente, maior acesso a esses fármacos, que, muitas vezes, são obtidos em suas próprias casas, ampliando o uso indevido entre os membros da família^(19)^.

O uso de substâncias entre adolescentes LGBT+ ainda é pouco explorado na literatura, embora represente um importante achado para entender as questões de saúde pública que envolvam essa população, como a vulnerabilidade social e os desafios psicossociais enfrentados. O atual estudo apontou que a prevalência da automedicação na vida foi quase duas vezes maior entre escolares LBGT+ em comparação aos heterossexuais. Essa relação se manteve para a automedicação atual, passando a ser 2,5 vezes mais prevalente entre eles. Tais resultados estão em consonância com uma análise de dados dos Estados Unidos que demonstrou que adolescentes de minorias sexuais, independentemente do sexo, apresentaram maior probabilidade de relatar uso indevido de esteroides anabolizantes androgênicos (EAAs) em níveis moderados ou graves quando comparados com seus pares heterossexuais^(20)^.

Nesse contexto, um fator a ser considerado é a teoria do estresse de minoria, que explica esse tipo de comportamento como uma resposta aos impactos do estigma social, da rejeição e da discriminação. Nesse contexto, o uso de substâncias pode ser um mecanismo de enfrentamento dos conflitos vividos por esses adolescentes^(21)^. Outro estudo internacional reforça esses achados, ao apontar que adolescentes do sexo masculino pertencentes a minorias sexuais apresentaram uma prevalência significantemente maior de uso indevido de EAAs ao longo da vida, atribuindo esse comportamento aos efeitos do estresse minoritário e à maior insatisfação corporal, fatores que podem atuar como motivadores e como forma de enfrentamento e busca por adequação a padrões estéticos ou de identidade de gênero^(22)^.

Ao analisar o atual estudo, verificou-se, na presente pesquisa, que adolescentes que possuíam algum vínculo de trabalho apresentavam maior prevalência de automedicação. Estudo realizado nos Estados Unidos com dados dos Inquéritos Nacionais de Saúde e Desenvolvimento Humano encontrou uma associação entre estar empregado e taxas aumentadas de uso recente e uso excessivo de substância entre adolescentes. Esse fenômeno pode ser explicado por vários fatores, como a inserção dos estudantes no mercado de trabalho, que os expõe a outros ciclos sociais com pessoas de diferentes idades, favorecendo o uso de substâncias ilícitas. O acesso a essas substâncias é facilitado pelo aumento da renda decorrente do trabalho. Outro fator apontado é o estresse e a sobrecarga de conciliar os estudos com o trabalho, o que pode levar o adolescente a buscar a automedicação como forma inadequada de enfrentar sintomas como fadiga, dificuldade para dormir e ansiedade^(23)^.

Este estudo apresenta algumas limitações. A primeira referese à natureza transversal do estudo, o que dificulta estabelecer relações de causalidade, uma vez que não é possível a determinação da sequência entre exposição e desfecho. Outra limitação é a possibilidade de viés de memória na obtenção dos dados. A coleta de informações se baseou em autorrelato, o que pode ter feito com que os participantes não se lembrassem do nome ou da classe dos medicamentos. Além disso, considerase o viés de informação, que pode ter levado à omissão de dados, seja pelo esquecimento ou pela relutância em declarar práticas socialmente não aceitas. Apesar disso, o questionário autopreenchido minimiza a relutância em declarar práticas não aceitas socialmente.

## CONCLUSÃO

Os dados apresentados no estudo evidenciaram uma prevalência importante de automedicação alguma vez na vida e automedicação atual entre adolescentes do ensino médio da RMGV. Maiores frequências foram observadas nas variáveis sexo feminino, escolares não cisgênero, LGBTQIA+, classificação socioeconômica mais elevada, aqueles que possuíam algum vínculo de trabalho e que referiram outras religiões além das católicas e evangélica. Desse modo, os achados reforçam a complexibilidade da automedicação entre os adolescentes, que envolve não apenas questões de acesso e disponibilidade de medicamentos, mas também aspectos psicossociais e culturais. Além disso, destacam o quanto o ambiente doméstico e social são fatores determinantes nesse processo de experimentação e uso inadequado de substâncias entre os escolares. Assim, os resultados desta pesquisa mostram a necessidade de estratégias intersetoriais que realizem ações de educação em saúde e promovam políticas públicas voltadas à promoção do uso racional de medicamentos entre os estudantes.

## Data Availability

Todo o conjunto de dados que dá suporte aos resultados deste estudo foi publicado no próprio artigo.

# Translation: Prevalencia y factores asociados a la automedicación entre escolares de la Región Metropolitana de Vitória, Brasil

**DOI:** 10.1590/1980-220X-REEUSP-2025-0395es

**Authors:** Kamila Rocha Mairink, Fernanda Garcia Gabira Miguez, Carlos Augusto Lopes, Franciele Foschiera Camboin, Nathalia Miguel Teixeira Santana, Nathalia Borba Raposo Pereira, Franciéle Marabotti Costa Leite

**Affiliations:** 1 Universidade Federal do Espírito Santo, Departamento de Enfermagem, Vitória, ES, Brasil.; 2 Universidade Federal do Espírito Santo, Programa de Pós-Graduação em Saúde Coletiva, Vitória, ES, Brasil.; 3 Universidade Federal do Espírito Santo, Programa de Ciências Sociais, Vitória, ES, Brasil.; 4 Universidade Estadual do Oeste do Paraná, Cascavel, PR, Brasil.; 5 Universidade Federal do Espírito Santo, Programa de Pós-Graduação em Psicologia, Vitória, ES, Brasil.

**Keywords:** Adolescente, Salud del Adolescente, Automedicación, Medicamentos sin Prescripción

## Abstract

**Objetivo:**

Analizar la prevalencia y los factores asociados con la automedicación en algún momento de la vida y su uso actual en estudiantes de secundaria de la Región Metropolitana de Gran Vitória, Espírito Santo.

**Método:**

Estudio transversal, realizado en escuelas entre marzo y diciembre de 2023 con 4416 estudiantes. Los resultados fueron la experimentación y el uso actual de medicamentos sin receta. Las variables categóricas independientes fueron sexo, grupo de edad, identidad de género, raza/color de piel, clasificación socioeconómica, tipo de escuela, si actualmente tienen trabajo, empleo o negocio, religión y estado civil de los padres. Los datos se analizaron mediante la prueba de chi-cuadrado de Pearson y la regresión de Poisson.

**Resultados:**

La prevalencia de automedicación a lo largo de la vida entre los escolares fue del 22,7% (IC del 95%: 21,5–23,9), con mayor prevalencia entre las mujeres (28,5%; IC del 95%: 26,8–30,3). Existe una mayor prevalencia de automedicación a lo largo de la vida y de automedicación actual entre las mujeres, el alumnado LGBT+, las personas de clase socioeconómica alta y las personas con empleo.

**Conclusión:**

Los hallazgos refuerzan la complejidad de la automedicación en adolescentes, que involucra no sólo cuestiones de acceso y disponibilidad de medicamentos, sino también aspectos psicosociales y culturales.

## INTRODUCCIÓN

El acceso a los medicamentos ha proporcionado a la sociedad beneficios que implican la prevención, el mantenimiento y la recuperación de la salud, contribuyendo, en general, a prolongar la vitalidad humana^(1)^. Sin embargo, a pesar de estos avances innegables, el uso irracional de medicamentos se configura como un problema de salud pública, ya que su uso inadecuado conlleva riesgos para la salud^(2)^.

Según la Organización Mundial de la Salud, más de la mitad de todos los medicamentos se dispensan, venden y recetan de forma incorrecta. Además, más del 50% de todos los países no aplican políticas básicas para promover su uso racional^(3)^. En Brasil, sin embargo, el uso incorrecto de los medicamentos está relacionado con la prescripción no orientada por directrices técnicas y clínicas establecidas por organismos reguladores y sociedades científicas, la automedicación inadecuada y la amplia disponibilidad de medicamentos para su compra en el mercado^(4)^.

Según una encuesta realizada por el Consejo Federal de Farmacia de Brasil, en 2019, el 77% de los brasileños consumieron medicamentos por su cuenta entre julio y diciembre. Este fenómeno también se da entre los jóvenes de 16 a 24 años, ya que alrededor del 69% de ellos alteran la posología del medicamento. Además, la facilidad de acceso a los medicamentos fue un factor determinante para la automedicación entre este grupo^(5)^.

Desde esta perspectiva, la creciente búsqueda del cuerpo perfecto en la sociedad contemporánea, asociada a la presión estética y a la internalización de estándares irreales, potenciada por el acceso a los medios digitales, crea un entorno propicio para la insatisfacción corporal entre los jóvenes. Así, la búsqueda de resultados rápidos contribuye significativamente a la toma de decisiones perjudiciales para la salud, como el uso de medicamentos adelgazantes y anabólicos entre los adolescentes^(6)^.

Otro problema es el uso de medicamentos narcóticos y psicoactivos entre los adolescentes. La primera experiencia con drogas y sustancias psicoactivas suele producirse en este grupo de edad. Esto pone de manifiesto la importancia de estudiar este fenómeno entre los jóvenes, ya que, movidos por la curiosidad y la influencia, estas sustancias se consumen de forma experimental^(7)^.

El Comité Nacional para la Promoción del Uso Racional de Medicamentos refuerza la importancia y la necesidad de tomar medidas para contener los abusos relacionados con el uso de medicamentos. Entre las recomendaciones estratégicas para alcanzar el objetivo propuesto se encuentran el fomento del debate sobre la problemática, la suma de esfuerzos con otros trabajos ya desarrollados y el estímulo de la producción científica en las áreas relacionadas^(8)^. Con este fin, el presente estudio tiene como objetivo analizar la prevalencia y los factores asociados a la automedicación alguna vez en la vida y su uso actual entre los estudiantes de secundaria de la Región Metropolitana de Gran Vitória (RMGV), Espírito Santo.

## MÉTODO

### Diseño del Estudio

Este estudio tiene un diseño transversal, realizado con datos de un estudio escolar titulado «Pesquisa estadual de saúde do escolar capixaba – PESC: uma análise dos escolares do ensino médio» (Estudio estatal sobre la salud de los escolares de Espírito Santo – PESC: un análisis de los estudiantes de secundaria), llevado a cabo entre marzo y diciembre de 2023, siendo el estudio piloto realizado en febrero del mismo año.

### Contexto

El estudio se llevó a cabo en la RMGV, compuesta por siete municipios del estado de Espírito Santo, Brasil, a saber: Vitória, Vila Velha, Serra, Cariacica, Viana, Guarapari y Fundão^(9)^. La población estimada en la RMGV para el año 2022 era de 1 880 828 personas, lo que representa alrededor del 49% de la población de Espírito Santo^(10)^. No hay datos específicos sobre la población adolescente en la RMGV, pero se estima que 63 255 alumnos están matriculados en la educación secundaria. Esta región cuenta con 171 escuelas de secundaria, entre públicas y privadas^(11)^.

### Participantes

El público de la investigación incluyó a estudiantes de secundaria de la RMGV. El proceso de muestreo se basó en los datos del Instituto Nacional de Estudios e Investigaciones Educativas Anísio Teixeira de 2020 para la selección de las escuelas, que se separaron según la proporción de estudiantes en cada estrato: red pública y red privada. Para cada estrato, se calculó una «N» utilizando un muestreo aleatorio simple, corregido para muestras complejas en un segundo momento utilizando el programa estadístico Statistical Package for the Social Sciences, 26.0, garantizando un 95% de confianza y un error máximo del 5%. Para los estratos municipales, se garantizaron como parámetros de la muestra un 95% de confianza y un error máximo del 10%, siendo la muestra mínima de 4416.

### Recolección de Datos

El cuestionario fue respondido directamente por el estudiante a través del Dispositivo Móvil de Recolección (tablet), en el que se insertó el cuestionario estructurado. La elección de este instrumento tuvo como objetivo agilizar el proceso de ejecución de la investigación, ya que, al ser una tecnología conocida, despierta un mayor interés de los adolescentes en participar en la recolección de datos, además de ser más fácil de manejar. Cabe destacar que solo participaron en la investigación los estudiantes con el consentimiento formal de sus tutores, mediante la firma del Término de Consentimiento Libre e Informado, y el consentimiento de los propios adolescentes, mediante el Término de Consentimiento Libre e Informado. Todos los participantes fueron informados previamente sobre los objetivos de la investigación, así como sobre sus posibles riesgos y beneficios.

## RESULTADOS

Los resultados estudiados fueron la automedicación alguna vez en la vida (sí/no) y la automedicación actual (sí/no). Los resultados se construyeron a partir del uso de medicamentos para adelgazar (Ritalina, Anfepramona, Femproporex, Mazindol, Hipofagin, Inibex, Desobesi, Moderine, Absten, Fagolipo y Dualid), anabólicos (Anabolex, Androlone, Androviron, Decadurabolin, Durabolin, Durateston, Equipoise y Parabolan), tranquilizantes (Diazepam, Dienpax, Valium, Lorax, Rohypnol, Psicosedin, Somalium, Apraz, Rivotril, Dormonid, Bromazepam, Frontal, Olcadil y Zolpidem) o estupefacientes (Morfina, Cetamina, Tylex, Setux, Sylador, Tramal, Dolantina, Fentanil y Belacodid).

### Independientes

Las variables independientes están compuestas por sexo (femenino/masculino), rango de edad (14 a 15, 16 a 17 o 18 a 19 años), identidad de género (cisgénero: sí o no), orientación sexual (heterosexual o LGBT+), raza/color de piel (blanca, negra o morena), estado civil (con o sin pareja), clasificación socioeconómica (A, B1, B2, C1, C2 o C3, D o E), tipo de escuela (pública o privada), si actualmente tiene algún trabajo, empleo o negocio (no o sí), religión (ninguna, católica, evangélica u otras) y situación conyugal de los padres (viven juntos: sí o no).

### Análisis de Datos

Los datos se presentaron primero mediante frecuencia absoluta, frecuencia relativa e intervalo de confianza del 95% (IC95%). Posteriormente, los datos se evaluaron mediante análisis bivariado, utilizando la prueba Chi-cuadrado de Pearson para establecer posibles relaciones, y mediante análisis multivariado bruto y ajustado, a través de la razón de prevalencia (RP), con varianza robusta por regresión de Poisson, considerándose para su inclusión en el ajuste las variables con significación estadística en 0,20 y manteniéndose aquellas con valor de p < 0,05. Los análisis de datos se realizaron con el programa estadístico Stata 17.0.

Esta investigación fue sometida al Comité de Ética en Investigación de la Universidad Federal de Espírito Santo y recibió la aprobación mediante el Dictamen n.° 5.900.370, lo que garantiza el cumplimiento de todos los requisitos éticos necesarios.

## RESULTADOS

Se observa que la prevalencia de la automedicación alguna vez en la vida fue del 22,7% (IC del 95%: 21,5–23,9) en la muestra general. Las estudiantes de sexo femenino (28.5%; IC 95%: 26.8–30.3) presentaron frecuencias más altas en comparación con las de sexo masculino (15.7%; IC 95%: 14.2–17.3). Además, el 7.7% (IC del 95%: 6.9–8.5) de los participantes refirieron automedicación actual, siendo también más prevalente entre las jóvenes (10.3%; IC del 95%: 9.2–11.6) que entre los muchachos (4.5%; IC del 95%: 3.7–5.4) (Tabla 1).

**Tabla 1:** Automedicación alguna vez en la vida y automedicación actual entre los estudiantes de secundaria de la Región Metropolitana de Grande Vitória – Espírito Santo, Brasil, marzo a diciembre, 2023 (N = 4613).

Variables	Muestra general			Femenina			Masculino		
	N	%	IC95%	N	%	IC95%	N	%	IC95%
Automedicación alguna vez en la vida									
No	3567	77,3	76,1–78,5	1790	71,5	69,7–73,2	1777	84,3	82,7–85,8
Sí	1046	22,7	21,5–23,9	715	28,5	26,8–30,3	330	15,7	14,2–17,3
Automedicación actual									
No	4260	92,4	91,5–93,1	2247	89,7	88,4–90,8	2013	95,5	94,6–96,3
Sí	353	7,7	6,9–8,5	258	10,3	9,2–11,6	94	4,5	3,7–5,4

*Un *missing* en el sexo masculino; N – frecuencia absoluta; % – frecuencia relativa; IC95% – Intervalo de Confianza del 95%.

En la Tabla 2 se observa que la automedicación alguna vez en la vida y la automedicación actual se relacionaron con las variables sexo, identidad de género, orientación sexual, estado civil, clase económica y religión. La variable situación conyugal de los padres solo se relacionó con la automedicación alguna vez en la vida, mientras que trabajar y tener algún trabajo, empleo o negocio se relacionó con la automedicación actual (p < 0,05).

**Tabla 2:** Distribución de la automedicación alguna vez en la vida y la automedicación actual entre los estudiantes de secundaria de la Región Metropolitana de Grande Vitória – Espírito Santo, Brasil, marzo a diciembre, 2023 (N = 4.613).

Variables	Automedicación en algún momento de la vida				Automedicación actual			
	N	%	IC95%	Valor de p	N	%	IC95%	Valor de p
**Sexo**				<0,001				<0,001
Femenino	715	28,5	26,8–30,3		258	10,3	9,2–11,6	
Masculino	330	15,7	14,2–17,3		94	4,5	3,7–5,4	
**Rango de edad**				0,168				0,405
14 a 15	213	22,0	19,5–24,7		84	8,7	7,0–10,6	
16 a 17	678	22,3	20,9–21,8		223	7,3	6,5–8,3	
18 a 19	155	25,7	22,3–29,3		46	7,6	5,8–10,0	
**Cisgénero**				<0,001				0,001
Sí	948	22,0	20,7–23,2		315	7,3	6,6–8,1	
No	96	32,9	27,8–38,5		37	12,7	9,3–17,7	
**Orientación sexual**				<0,001				<0,001
Heterosexual	700	19,1	17,9–20,4		215	5,9	5,2–6,7	
LGBT+	345	36,4	33,4–39,5		137	14,4	12,3–16,8	
**Raza/color de piel**				0,726				0,098
Blanca	387	21,8	19,9–23,8		125	7,0	5,9–8,3	
Negra	173	22,8	19,9–25,9		71	9,3	7,5–11,6	
Moreno	445	22,8	21,0–24,8		139	7,1	6,1–8,4	
**Estado civil**				<0,001				0,045
Con pareja	324	26,4	24,0–28,9		243	7,2	6,4–8,1	
Sin pareja	722	21,3	20,0–28,9		110	9,0	7,5–10,7	
**Clasificación socioeconómica**				0,001				0,001
A	212	27,8	24,7–31,2		212	27,8	24,7–31,1	
B1/B2	465	21,4	19,7–23,1		465	21,4	19,8–23,1	
C1/C2/D/E	367	22,0	20,1–24,1		367	22,0	20,1–24,1	
**Tipo de escuela**				0,087				0,140
Pública	678	21,9	20,5–23,4		224	7,3	6,4–8,2	
Privada	368	24,2	22,1–26,4		129	8,5	7,2–10,0	
**Actualmente tiene algún trabajo, empleo o negocio**				0,054				0,012
No	769	22,0	20,7–23,4		248	7,11	6,3–8,0	
Sí	277	24,8	22,3–27,4		105	9,4	7,8–11,2	
**Religión**				<0,001				<0,001
Ninguna	268	24,6	22,1–27,3		95	8,7	7,2–10,6	
Católica	242	20,5	18,3–22,9		79	6,7	5,4–8,3	
Evangélico	406	20,2	18,5–22,0		132	6,6	5,6–7,7	
Otros	130	38,5	33,4–43,8		47	13,9	10,7–18,0	
**Situación marital de los padres: viven juntos**				0,036				0,455
Sí	517	21,4	19,8–23,1		178	7,4	6,4–8,5	
No	519	24,0	22,2–25,8		172	8,0	6,9–9,2	

N – frecuencia absoluta; % – frecuencia relativa; IC95% – Intervalo de confianza del 95%.

Tras ajustar los factores de confusión, se observó que las estudiantes del sexo femenino presentaban una prevalencia un 67% mayor de automedicación alguna vez en la vida en comparación con los del sexo masculino (RPa = 1,67; IC95%: 1,48–1,89; p < 0,001). Además, se observó una mayor frecuencia en el grupo de edad de 18 a 19 años (RPa = 1,24; IC95%: 1,04–1,48; p = 0,044) cuando s l comparar con el grupo de 14 y 15 años. Del mismo modo, los participantes LGBTQIA+ (RPa: 1,55), de clasificación socioeconómica más alta (RPa: 1,36), que declararon seguir otras religiones que no fueran evangélicas y católicas (RPa: 1,50), y aquellos que tenían trabajo, empleo o negocio (RPa: 1,15) presentaron una mayor prevalencia de automedicación alguna vez en la vida (Tabla 3).

**Tabla 3:** Razón de prevalencia bruta y ajustada de la automedicación alguna vez en la vida entre los estudiantes de secundaria de la Región Metropolitana de Grande Vitória – Espírito Santo, Brasil, marzo a diciembre, 2023.

Variables	Automedicación en algún momento de la vida					
	RP bruta	IC95	Valor de p	RP ajustada	IC95%	Valor de p
**Sexo**			<0,001			<0,001
Femenino	1,82	1,62–2,05		1,67	1,48–1,89	
Masculino	1,0			1,0		
**Rango de edad**			0,159			0,044
14 a 15	1,0			1,0		
16 a 17	1,02	0,89–1,16		1,05	0,92–1,20	
18 a 19	1,17	0,98–1,40		1,24	1,04–1,48	
**Cisgénero**			<0,001			0,173
Sí	1,0			1,0		
No	1,50	1,26–1,78		1,13	0,95–1,35	
**Orientación sexual**			<0,001			<0,001
Heterosexual	1,0			1,0		
LGBT+	1,90	1,71–2,12		1,55	1,37–1,74	
**Raza/color de piel**			0,726			–
Blanca	1,0			–	–	
Negra	1,04	0,89–1,22		–	–	
Moreno	1,04	0,93–1,18		–	–	
**Estado civil**			<0,001			0,159
Con pareja	1,24	1,10–1,39		1,09	0,97–1,74	
Sin pareja	1,0			1,0		
**Clasificación socioeconómica**			0,001			<0,001
A	1,26	1,09–1,46		1,36	1,18–1,57	
B	0,97	0,86–1,09		1,01	0,90–1,14	
C//D/E						
**Tipo de escuela**			0,086			0,338
Pública	1,0			1,0		
Privada	1,10	0,99–1,23		1,06	0,94–1,21	
**Actualmente tiene algún trabajo, empleo o negocio**			0,052			0,018
No	1,0			1,0		
Sí	1,13	1,00–1,27		1,15	1,02–1,29	
**Religión**			<0,001			<0,001
Ninguna	1,0			1,0		
Católica	0,83	0,72–0,97		0,95	0,81–1,11	
Evangélica	0,82	0,72–0,94		0,96	0,83–1,10	
Otras	1,56	1,32–1,85		1,50	1,27–1,77	
**Situación marital de los padres: viven juntos**			0,036			0,241
Sí	1,0			1,0		
No	1,12	1,01–1,25		1,07	0,96–1,91	

RP: razón de prevalencia; IC95%: intervalo de confianza del 95%.

En el análisis ajustado, la prevalencia actual de la automedicación en las mujeres fue aproximadamente dos veces mayor (RPa = 2,12; IC95%: 1,65–2,72; p < 0,001) que en los hombres. Los estudiantes LGBTQIA+ también presentaron frecuencias más altas (RPa = 2,04; IC95%: 1,64–2,54; p < 0,001) en comparación con los heterosexuales. En cuanto a la raza/color de piel, los adolescentes que se declararon negros presentaron una mayor prevalencia de automedicación actual (RPa: 1,49), y esta práctica fue dos veces mayor entre los estudiantes de clase económica «A» (RPa = 2,27; IC95%: 1,73–2,97; p < 0,001) y se observó con mayor frecuencia entre los que tenían trabajo (RPa = 1,39; IC95%: 1,12–1,74; p = 0,003) (Tabla 4).

**Tabla 4:** Razón de prevalencia bruta y ajustada de la automedicación actual entre los estudiantes de secundaria de la Región Metropolitana de Grande Vitória – Espírito Santo, Brasil, marzo a diciembre, 2023.

Variables	Automedicación actual					
	RP bruto	IC95	Valor de p	RP ajustada	IC95%	Valor de p
**Sexo**			<0,001			<0,001
Femenino	2,31	1,84–2,90		2,12	1,65–2,72	
Masculino	1,0			1,0		
**Rango de edad**			0,404			–
14 a 15	1,0			–	–	
16 a 17	0,85	0,67–1,08		–	–	
18 a 19	0,88	0,63–1,24		–	–	
**Cisgénero**			0,001			0,594
Sí	1,0			1,0		
No	1,74	1,26–2,39		1,10	0,78–1,56	
**Orientación sexual**			<0,001			<0,001
Heterosexual	1,0			1,0		
LGBT+	2,50	2,01–3,01		2,04	1,64–2,54	
**Raza/color de piel**			0,097			0,019
Blanca	1,0			1,0		
Negra	1,33	1,00–1,75		1,49	1,13–1,96	
Moreno	1,01	0,80–1,28		1,13	0,90–1,42	
**Estado civil**			0,044			0,930
Con pareja	1,25	1,01–1,55		0,99	0,79–1,25	
Sin compañero	1,0			1,0		
**Clasificación socioeconómica**			<0,001			<0,001
A	1,85	1,42–2,40		2,27	1,73–2,97	
B	1,04	0,82–1,32		1,20	0,94–1,54	
C//D/E	1,0			1,0		
**Tipo de escuela**			0,140			0,649
Pública	1,0			1,0		
Privada	1,20	0,95–1,44		1,06	0,82–1,37	
**Actualmente tiene algún trabajo, empleo o negocio**			0,012			0,003
No	1,0			1,0		
Sí	1,32	1,06–1,65		1,39	1,12–1,74	
**Religión**			<0,001			0,129
Ninguna	1,0			1,0		
Católica	0,77	0,58–1,02		0,90	0,66–1,22	
Evangélica	0,75	0,58–0,97		0,90	0,68–1,18	
Otras	1,59	1,15–2,21		1,32	0,94–1,86	
**Situación marital de los padres: viven juntos**			0,455			–
Sí	1,0			–	–	
No	1,08	0,88–1,32		–	–	

RP – Razón de prevalencia; IC95% – Intervalo de confianza del 95%.

## DISCUSIÓN

El presente estudio muestra que un número significativo de adolescentes ha consumido medicamentos sin receta médica alguna vez en su vida y lo sigue haciendo en la actualidad. Este resultado corrobora los hallazgos nacionales que refuerzan la alta prevalencia del consumo de medicamentos entre los jóvenes. Una investigación transversal realizada en Porto Alegre reveló que aproximadamente la mitad de los estudiantes declaró haber consumido medicamentos en los siete días previos a la recolección de datos. Parte de este consumo puede estar asociado a la práctica de la automedicación, que se ve facilitada por el fácil acceso a medicamentos sin receta médica, adquiridos directamente en farmacias o encontrados en el botiquín del hogar. A este problema se suma la exposición a campañas que resultan perjudiciales, ya que promueven y estimulan el consumo, incluso en ausencia de orientación profesional, a personas que pueden no tener una postura crítica hasta el punto de reconocer los límites de su uso^(12)^.

Otro factor que justifica esta conducta entre los escolares es el conocimiento limitado sobre el uso racional de los medicamentos. La escasa comprensión de las indicaciones, las dosis y la aplicación del fármaco se ha señalado como uno de los factores determinantes de comportamientos de riesgo, como practicar la polifarmacia, tomar medicamentos en dosis excesivas y no leer el prospecto. Un estudio internacional reforzó esta comprensión al demostrar que los adolescentes con un bajo nivel de conocimiento sobre los medicamentos presentaban una mayor prevalencia de automedicación^(13)^. Además, la forma en que los adolescentes perciben su propia salud influye directamente en sus prácticas de cuidado. Los adolescentes que tienen una mala percepción de su salud tienden a utilizar más medicamentos de diferentes clases terapéuticas, lo que refuerza la asociación entre la percepción negativa de la salud y un mayor consumo de medicamentos^(14)^.

El ciclo de amistades se configuró como uno de los principales medios de acceso a tranquilizantes y sedantes no recetados entre los adolescentes^(15)^. La búsqueda de sensaciones y efectos recreativos se asoció con la adquisición de estos medicamentos a través de amigos, siendo esta práctica de compartir entre pares común, especialmente entre jóvenes que ya han tenido recetas legítimas. De este modo, la vulnerabilidad evidenciada por la falta de conocimiento sobre los riesgos del uso descontrolado de estos fármacos pone de manifiesto la gravedad del uso de sustancias de alto riesgo entre los escolares^(16)^.

Las mayores prevalencias de automedicación, ya sea alguna vez en la vida o actualmente, se observaron en adolescentes de sexo femenino. Un estudio de cohorte realizado en Pelotas constató que las mujeres consumen significativamente más medicamentos que los hombres, incluso cuando se excluyen los anticonceptivos de los análisis^(17)^. De manera similar, los patrones internacionales reafirman esta diferencia entre los géneros. Un análisis transversal internacional, realizado en España con estudiantes entre 2016 y 2021, observó que el uso no prescrito de tranquilizantes, como benzodiazepínicos e hipnóticos, entre las chicas era aproximadamente el doble que entre los chicos. Además, las variables asociadas de forma independiente y significativa a esta mayor probabilidad fueron el consumo de alcohol y *cannabis* en los últimos 30 días^(18)^.

En este sentido, la incorporación de una nueva perspectiva de género en el consumo de sustancias psicoactivas permite ampliar la comprensión de los diferentes patrones de consumo entre hombres y mujeres. Entre las causas se encuentran la mayor incidencia de cuadros depresivos y ansiosos entre las mujeres, incluso en la adolescencia, y su posición desigual en relación con los hombres. También se considera que esta población tiende a percibir y reportar sus síntomas con mayor frecuencia, lo que la lleva a buscar más los servicios de salud y a contribuir a un proceso de medicalización de sus experiencias emocionales. Posteriormente, utilizan estos medicamentos con alteraciones en la posología^(18)^.

Otro hallazgo de la presente investigación es la mayor prevalencia de la automedicación alguna vez en la vida y actualmente entre los adolescentes de clase económica A. Estos resultados corroboran una investigación nacional transversal, realizada en las 27 capitales brasileñas con estudiantes de secundaria de escuelas públicas y privadas, que evidencia el uso no prescrito de tranquilizantes/sedantes y que es más común entre aquellos con clases sociales más altas^(15)^.

Una posible hipótesis para explicar este hallazgo es que las personas en situaciones económicas más favorables tienen más acceso a los servicios de salud y, en consecuencia, mayor acceso a estos fármacos, que a menudo se obtienen en sus propios hogares, lo que aumenta el uso indebido entre los miembros de la familia^(19)^.

El uso de sustancias entre los adolescentes LGBT+ aún está poco explorado en la literatura, aunque representa un hallazgo importante para comprender las cuestiones de salud pública que afectan a esta población, como la vulnerabilidad social y los retos psicosociales a los que se enfrentan. El presente estudio señaló que la prevalencia de la automedicación a lo largo de la vida fue casi dos veces mayor entre los estudiantes LBGT+ en comparación con los heterosexuales. Esta relación se mantuvo para la automedicación actual, pasando a ser 2,5 veces más prevalente entre ellos. Estos resultados concuerdan con un análisis de datos de Estados Unidos que demostró que los adolescentes de minorías sexuales, independientemente de su sexo, tenían más probabilidades de informar el uso indebido de esteroides anabólicos androgénicos (EAA) en niveles moderados o graves en comparación con sus pares heterosexuales^(20)^.

En este contexto, un factor a tener en cuenta es la teoría del estrés minoritario, que explica este tipo de comportamiento como una respuesta a los impactos del estigma social, el rechazo y la discriminación. En este contexto, el uso de sustancias puede ser un mecanismo para afrontar los conflictos que viven estos adolescentes^(21)^. Otro estudio internacional refuerza estos hallazgos al señalar que los adolescentes varones pertenecientes a minorías sexuales presentaron una prevalencia significativamente mayor de uso indebido de EAA a lo largo de la vida, atribuyendo este comportamiento a los efectos del estrés minoritario y a una mayor insatisfacción corporal, factores que pueden actuar como motivadores y como forma de afrontamiento y búsqueda de adecuación a los estándares estéticos o de identidad de género^(22)^. Al analizar el presente estudio, se observó que los adolescentes que tenían algún vínculo laboral presentaban una mayor prevalencia de automedicación.

Un estudio realizado en Estados Unidos con datos de las Encuestas Nacionales de Salud y Desarrollo Humano encontró una asociación entre estar empleado y el aumento de las tasas de consumo reciente y excesivo de sustancias entre los adolescentes. Este fenómeno puede explicarse por varios factores, como la inserción de los estudiantes en el mercado laboral, que los expone a otros círculos sociales con personas de diferentes edades, lo que favorece el consumo de sustancias ilícitas. El acceso a estas sustancias se ve facilitado por el aumento de los ingresos derivados del trabajo. Otro factor señalado es el estrés y la sobrecarga que supone conciliar los estudios con el trabajo, lo que puede llevar al adolescente a buscar la automedicación como forma inadecuada de afrontar síntomas como la fatiga, la dificultad para dormir y la ansiedad^(23)^.

Este estudio presenta algunas limitaciones. La primera se refiere a la naturaleza transversal del estudio, lo que dificulta establecer relaciones de causalidad, ya que no es posible determinar la secuencia entre la exposición y el resultado. Otra limitación es la posibilidad de sesgo de memoria en la obtención de los datos. La recolección de información se basó en el autorrelato, lo que puede haber hecho que los participantes no recordaran el nombre o la clase de los medicamentos. Además, se considera el sesgo de información, que puede haber llevado a la omisión de datos, ya sea por olvido o por renuencia a declarar prácticas socialmente inaceptables. A pesar de ello, el cuestionario autoadministrado minimiza la renuencia a declarar prácticas socialmente inaceptables.

## CONCLUSIÓN

Los datos presentados en el estudio evidenciaron una prevalencia importante de automedicación alguna vez en la vida y automedicación actual entre los adolescentes de secundaria de la RMGV. Se observaron mayores frecuencias en las variables de sexo femenino, estudiantes no cisgénero, LGBTQIA+, clasificación socioeconómica más alta, aquellos que tenían algún vínculo laboral y que referían otras religiones además de la católica y la evangélica. De este modo, los hallazgos refuerzan la complejidad de la automedicación entre los adolescentes, que implica no solo cuestiones de acceso y disponibilidad de medicamentos, sino también aspectos psicosociales y culturales. Además, destacan la importancia del entorno doméstico y social como factores determinantes en este proceso de experimentación y uso inadecuado de sustancias entre los estudiantes. Así, los resultados de esta investigación muestran la necesidad de estrategias intersectoriales que lleven a cabo acciones de educación en salud y promuevan políticas públicas orientadas a fomentar el uso racional de medicamentos entre los estudiantes.

## Data Availability

Todo el conjunto de datos que apova los resultados de este estudio fue publicado en el propio artículo.
